# Distribution of Tetrodotoxin in the New Zealand Clam, *Paphies australis*, Established Using Immunohistochemistry and Liquid Chromatography-Tandem Quadrupole Mass Spectrometry

**DOI:** 10.3390/toxins10070282

**Published:** 2018-07-06

**Authors:** Laura Biessy, Kirsty F. Smith, Michael J. Boundy, Stephen C. Webb, Ian Hawes, Susanna A. Wood

**Affiliations:** 1 Cawthron Institute, Private Bag 2, Nelson 7010, New Zealand; kirsty.smith@cawthron.org.nz (K.F.S.); michael.boundy@cawthron.org.nz (M.J.B.); steve.webb@cawthron.org.nz (S.C.W.); susie.wood@cawthron.org.nz (S.A.W.); 2 Department of Biological Sciences, University of Waikato, Private Bag 3105, Hamilton 3240, New Zealand; ian.hawes@waikato.ac.nz

**Keywords:** biotoxin, localization, marine bivalves, monoclonal antibody, neurotoxin

## Abstract

Tetrodotoxin (TTX) is one of the most potent neurotoxins known. It was originally thought to only occur in puffer fish but has now been identified in twelve different classes of freshwater and marine organisms, including bivalves. Despite being one of the world’s most studied biotoxins, its origin remains uncertain. There is contradictory evidence regarding the source of TTX and its pathway through food webs. To date, the distribution of TTX has not been examined in bivalves. In the present study, 48 *Paphies australis*, a TTX-containing clam species endemic to New Zealand, were collected. Thirty clams were dissected, and organs and tissues pooled into five categories (siphons, digestive gland, adductor muscles, and the ‘rest’) and analyzed for TTX using liquid chromatography-mass spectrometry (LC-MS). The micro-distribution of TTX was visualized in the remaining 18 individuals using an immunohistological technique incorporating a TTX-specific monoclonal antibody. The LC-MS analysis revealed that siphons contained the highest concentrations of TTX (mean 403.8 µg/kg). Immunohistochemistry analysis showed TTX in the outer cells of the siphons, but also in the digestive system, foot, and gill tissue. Observing TTX in organs involved in feeding provides initial evidence to support the hypothesis of an exogenous source in *P. australis*.

## 1. Introduction

Tetrodotoxin (TTX) is a lethal neurotoxin that selectively binds and blocks voltage-gated sodium channels [1]. It is one of the most potent natural substances known, with ingestion of 1 to 2 mg enough to kill a 50-kg human [2]. Tetrodotoxin was named after the puffer fish family Tetraodontidae and was first isolated in 1910 [3], although characterization of the chemical structure was not achieved until 1964 [4,5,6].

Tetrodotoxin has long been known as the causative agent in puffer fish poisoning [7,8] and was first recorded in 1774 by Captain James Cook who detailed the effects on his crew from the consumption of a fish from New Caledonia, now thought to be a puffer fish [9]. It was originally thought that TTX only occurred in puffer fish, but has since been identified in 12 different classes of marine, freshwater, and terrestrial vertebrate and invertebrate organisms [10].

Despite TTX being one of the most studied biotoxins in the world, its origin remains uncertain. There is contradictory evidence regarding whether the source of TTX is exogenous or endogenous, and the pathways and mechanisms through which TTX is incorporated in the food web are unknown [11]. The wide distribution of TTX in many genetically unrelated species and its high spatiotemporal variation among TTX-containing species suggest that the toxin comes from an exogenous source such as accumulation through diet or symbiotic bacteria [12,13,14]. However, there is also evidence for an endogenous source in terrestrial species. Research suggesting this began in the late 1980s when TTX-containing tree frogs (*Atelopus oxyrhynchus*) were shown to retain high levels of toxicity in controlled environment for three years [15]. Hatched frogs (*Atelopus varius*) raised in captivity were also found to contain TTX [16]. Similar experiments undertaken in newts (*Taricha granulosa*) have shown that individuals regenerated their levels of TTX in their skin when kept in captivity and fed a TTX-free diet [17].

Tetrodotoxin has been identified in nine different bivalve species from six countries. The first report of TTX in marine bivalves was in Japan in 1993 [18] when the toxin was found in the digestive gland of scallop (*Patinopecten yessoensis*) after a bloom of the dinoflagellate *Alexandrium tamarense*. Two decades after the first detection in marine bivalves, McNabb et al. [19] reported high levels (800 µg/kg) of TTX in the surf clam *Paphies australis* in New Zealand. That research triggered further investigations on TTX in bivalves globally. Turner et al. [20] detected TTX in Pacific oysters (*Crassostrea gigas*) and common blue mussels (*Mytilus edulis*) in England. Shortly after, Vlamis et al. [21] reported moderate levels (up to 223 µg/kg) of TTX in *M. edulis* harvested in 2012 from the Greek islands. Oysters and mussels from production areas in the Netherlands also tested positive for TTX [22] and trace detections (2.22 µg/kg) have also been reported in a clam species (*Ruditapes philippinarum*) in China [23].

Recent advances in chemical detection and quantification methods (i.e., Liquid Chromatography-Tandem Quadrupole Mass Spectrometry; LC-MS/MS) have allowed the development of very specific, sensitive, and accurate methods to measure TTX concentrations [24,25]. The use of these methods makes it possible to investigate and quantify the distribution of TTX in specific organs within bivalves. To date, the micro-distribution of TTX has been studied in a wide range of organisms including: three species of pufferfish [26,27]; a sea-slug and a flatworm [28]; a nemertean and a turbellarian [29]; and a ribbon worm [30] but has never been investigated in bivalves. Improving knowledge on the location of TTX in marine bivalves may provide new insights on the ecological functions and sources of the neurotoxin in these organisms. For example, finding the toxin in the outside epithelium or in the reproductive system could indicate a defense mechanism, whereas the identification of TTX in the digestive tract could indicate a possible exogenous source.

In the present study, LC-MS/MS was used to determine which *P. australis* organs contained TTX. These data were then used to guide immunohistological experiments using a TTX-specific monoclonal antibody [31,32] to determine the micro-distribution of TTX within these organs. Based on similar studies on the neurotoxin saxitoxin (produced by planktonic marine dinoflagellates and freshwater cyanobacterial species) in clams *Saxidomus gigantea* [33] and in other *Paphies* species [34], we hypothesized that TTX would be located in the siphons where it would act as a defense mechanism, and in the reproductive system of *P. australis* where it would be transferred to larvae to aid in their protection post-spawning.

## 2. Results and Discussion

Tetrodotoxin was the most abundant analogue in the matrices analyzed by LC-MS/MS, accounting for >99% of the total TTX analogues present. Traces of 4/6-epi-TTX, 4,9-anhydro-TTX, and two deoxy-TTX analogues, putative 5-deoxy-TTX and 11-deoxy-TTX, were detected but concentrations were too low to allow accurate quantification (Figure S1).

### 2.1. Siphons

**Figure 1:**
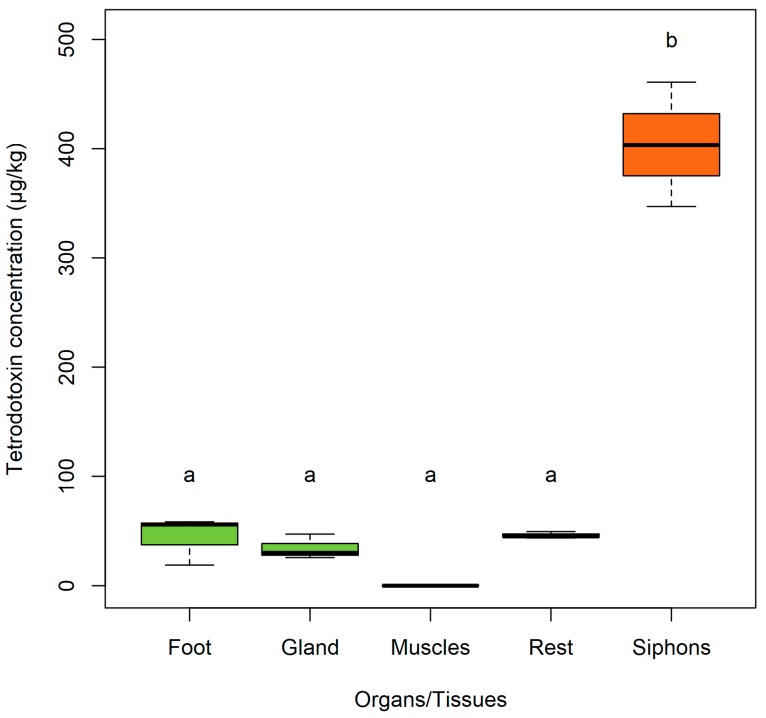
Tetrodotoxin concentrations in the organs and tissues of *Paphies australis* determined using liquid chromatography-mass spectrometry. Solid black line shows median, box shows 1st and 3rd quartiles, whiskers extend to the last data point within 1.5 times the inter-quartile range if there is data that far from it. Letters indicate where significant differences (one-way ANOVA with Tukey’s post-hoc test, *p* < 0.001) occur between organs/tissues.

**Figure 2:**
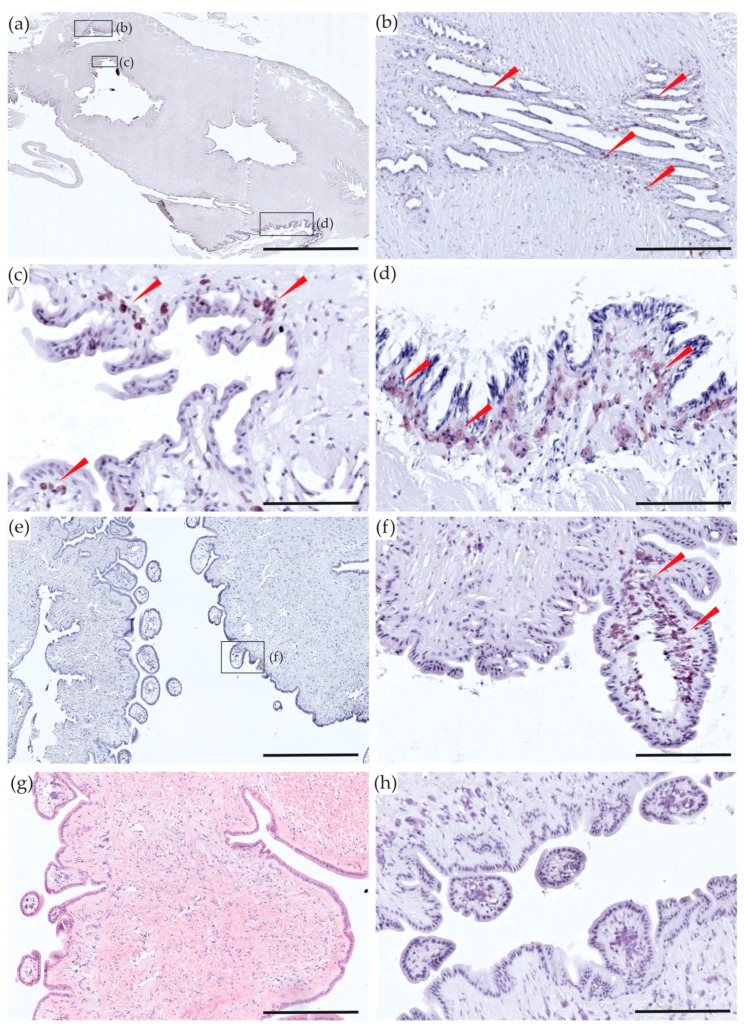
Longitudinal sections of the inhalant and exhalant siphons of *Paphies australis*. (**a**) Tetrodotoxin (TTX)-specific monoclonal antibody (mAB) immunohistological staining in longitudinal section of siphons; (**b**–**d**) enlargements of different boxes on (**a**) to show micro-location of TTX staining, identified by the brown color deposits shown by red arrows; (**e**) another view of TTX-specific mAB immunohistological staining in the outer layer of the siphon; (**f**) enlargement of the box on (**e**) to show detailed view of the staining in the siphons loops; (**g**) Hematoxylin and Eosin staining; (**h**) mAB negative control. Scale bars = 200 µm (**a**), 50 µm (**b**–**d**,**f**,**h**), 150 µm (**e**), and 100 µm (**g**).

Quantification of TTX in *P. australis* organs by LC-MS/MS identified the highest concentration of toxin in the siphons (mean 403.8 ± 32.8 µg/kg, Figure 1). One-way ANOVA showed a significant difference between the mean concentrations in organ groups (*p* < 0.001), with Tukey’s post-hoc test identifying the difference between the siphons and all other groups (*p* < 0.001; Figure 1). The identification of high concentrations in the siphon is similar to studies which showed that butter clams *S. gigantea* and the surf-clam *Paphies subtriangulata* sequester saxitoxin, in their siphons [33,34,35]. The immunostaining provided further evidence for the location of accumulation of TTX in the siphon sections. It was visualized as small brown deposits in the outside and inside layers of cells (Figure 2a–f). Slides stained with eosin and hematoxylin were used to identify organs and tissues but were also used to identify brown cells or rhogocytes ([36]; Figure 2g), to demonstrate that they had not been misinterpreted as TTX-specific staining. No TTX-like staining was observed in control slides not stained with anti-TTX, confirming that there was no confusion with brown cells (Figure 2h).

Bivalve siphons were an evolutionary development which enabled them to diversify into many new ecological niches after the Paleozoic Era [37]. In bivalves, the two siphons are paired: the inhalant siphon carries the water into the mantle cavity, and the exhalant siphon ejects the water from the internal cavity [38]. The water flow from the siphons is variously used for feeding, respiration, reproduction, and in locomotion for some bivalves [38]. Siphons also play a critical role in survival by allowing water flow while buried [39]. *Paphies australis* and many other bivalves, bury themselves in the sand and keep their siphons above the sediment surface to filter feed. It is well known that siphons are a targeted food for predators including siphon-nipping fish such as the flatfish *Pleuronectes platessa* [40] or shrimp [41]. Bivalves are negatively impacted by siphon-nipping predators because they require extra energy for siphon regeneration and it reduces feeding opportunities [41]. Researchers have suggested previously that biotoxins are accumulated as a chemical defense against predation. For example, Kvitek [35] suggested that the clam *S. gigantea* retains saxitoxin in their siphons as a defense mechanism against siphon-nipping predators and Kvitek & Bretz [42] demonstrated that sea otters stop grazing on shellfish with high saxitoxin concentrations. We speculate that TTX accumulation in siphon tissues could provide a similar chemical defense in *P. australis*. An elevated level of TTX in *P. australis* siphons could also suggest that TTX is present in the water or within organisms in the water that they are filtering while feeding, possibly indicating that the toxin comes from an exogenous source.

### 2.2. Foot

After the Palaeozoic era, certain bivalve species developed an extensive foot, opening up new life history strategies [37]. The development of a foot gave bivalves a distinct competitive advantage by allowing them to rapidly dig down into the sand [43]. Some are also able to swim short distances using their foot [38]. The LC-MS/MS analysis identified lower concentrations of TTX in foot tissue (mean 44.4 ± 12.8 µg/kg, Figure 1) but no TTX was vizualized after immunostaining. In *P. australis*, the foot is directly attached to the gonads. The toxic *P. australis* collected for this experiment were relatively small (<1.5 g) and not mature, hence the difficulty in separating the small gonads from the foot during the dissection. The LC-MS/MS detection is most likely ‘contamination’ from other organs. However, previous studies have detected low levels of saxitoxin in the foot of other bivalves including different clam species [44,45]. Pereira et al. [46] also detected small amounts of saxitoxin in the foot of freshwater mussels *Anodonta cygnea* after two days of exposure to the toxin.

### 2.3. Adductor Muscles

Adductor muscles are a significant part of the muscular system in bivalves, they are strong and connect the two valves [38]. Swimming movements of bivalves (in swimming species such as *Pecten* spp.) are produced by a series of sudden shell valve closures powered by rapid cycles of contraction and relaxation of the adductor muscles [47]. In this study, the muscle group included the posterior and anterior adductors muscles. No TTX was detected in the muscle using either immunostaining or LC-MS/MS quantification. Previous studies have shown that accumulation of marine biotoxins in the muscles of bivalves is very low or non-existent [48]. To take advantage of the low toxicity of this tissue in regions of the world where saxitoxin is present, sushi is now being made with bivalve adductor muscles [44].

### 2.4. Digestive System

**Figure 3:**
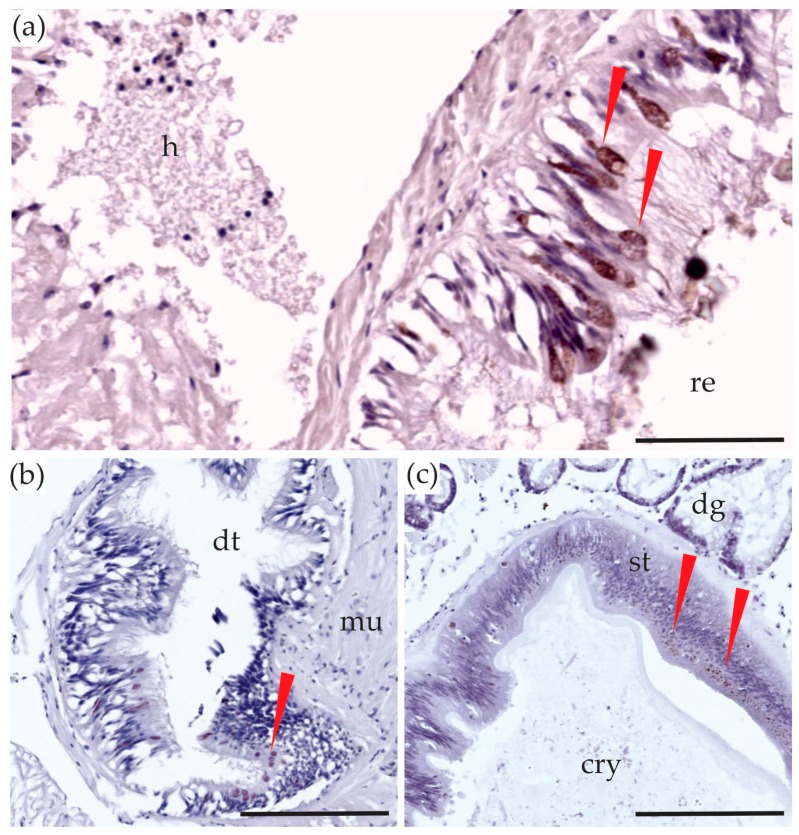
Sections of the digestive system of *Paphies australis*. Tetrodotoxin-specific monoclonal antibody immunohistological staining in: (**a**) the rectum; (**b**) the intestinal epithelium; (**c**) the inside layer of the stomach wall. The red arrows are showing the brown color deposits, indicating the presence of tetrodotoxin staining. cry = crystalline style, dg = tubules in the digestive gland, dt = lumen of the digestive tract, h = haemocyte debris, mu = muscle tissue, re = rectum. Scale bars = 50 µm (**a**) and 100 µm (**b**,**c**).

In bivalves, a short oesophagus leads from the mouth to the stomach. The stomach is surrounded by the digestive gland and an opening from the stomach leads to the intestine that extends into the foot, ending in the rectum and eventually the anus [49]. In this study, the digestive system consisted of the digestive gland, the oesophagus and the gut when it was large enough to identify and dissect. Tetrodotoxin was detected in the digestive system of the *P. australis* using LC-MS/MS (mean 34.3 ± 6.5 µg/kg; Figure 1) and was also observed in the inside cells of the epithelium of the intestine and the rectum in the immunostained sections (Figure 3). A number of studies have identified biotoxins in the digestive tracts of bivalve species, for example, the highest concentrations of saxitoxin was found in the viscera of *M. edulis* [50]; domoic acid was mostly contained (>80%) in mussel and oyster’s digestive systems [51] and greater than 50% of microcystins were present in the digestive glands of freshwater mussels [52]. This result could indicate that TTX is sourced from the diet of *P. australis*. Tetrodotoxin may be initially stored in the organs of the digestive system and could be transported to other tissues and organs, to use as a chemical defense.

### 2.5. The ‘rest’

The ‘rest’ group contained all the organs and tissues that were not described above. It mainly included the gonads, the mantle, the labial palps, and the gills. The separation of these tissues was not possible due to the fragility and small size of the *P. australis* collected. The ‘rest’ contained low concentrations of TTX (mean 46.1 ± 1.8 µg/kg; Figure 1). Although these sections could not be dissected for LC-MS/MS analysis, they could be visualised using the immunohistological staining and key findings are described below.

#### 2.5.1. Gonads

**Figure 4:**
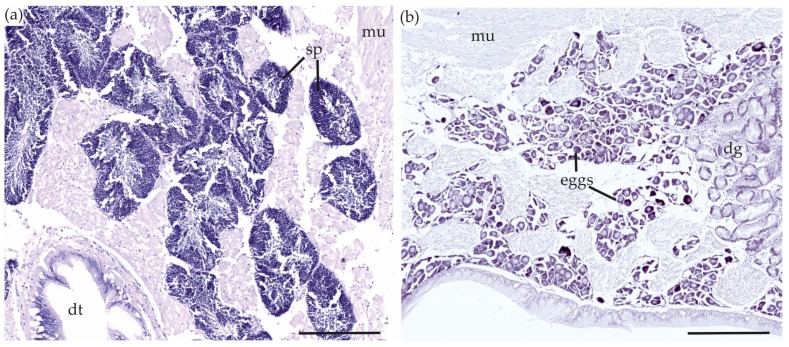
Gonads of *Paphies australis* turned dark purple after hematoxylin staining, but no evidence of TTX. (**a**) Male gonads; (**b**) Female gonads. dg = tubules in the digestive gland, dt = lumen of digestive tract, mu = muscle tissue, sp = sperm. Scare bars = 100 µm.

Like most bivalves, *P. australis* are dioecious and fertilization usually occurs in the water column after spawning [38]. In immunostained sections of *P. australis*, the gonads did not contain TTX. After hematoxylin staining, the eggs were dark purple (Figure 4), making the vizualisation of TTX challenging. Based on the immunostained sections, we do not believe TTX was present in the eggs. The majority of the *P. australis* collected for this study were not sexually mature (<40 mm shell length) and this may have affected our results if TTX migrates to the gonads only at sexual maturity.

Studies on other marine organisms have shown the presence of TTX in reproductive organs (e.g., in the gonads of the sea-slug *Pleurobranchaea maculata* and the flatworm *Stylochoplana* sp. [28]), and on the body surface of larvae from the pufferfish *Takifugu alboplumbeus* [53]. Further fine-scale studies are required to determine if *P. australis* eggs contain TTX using sexually mature individuals, and rearing studies could be used as the presence in mature eggs has significance to whether TTX is passed onto successive generations.

#### 2.5.2. Mantle

The mantle of the bivalves has a sensory function and can initiate closure of the valves in response to unfavourable conditions. It also has a respiratory function by controlling the inflow of water [49]. No TTX was visualized in the mantle after the immunostaining experiment. These results correlate with other studies investigating biotoxin distribution in bivalves. Studies by Pereira et al. [46] and Harada et al. [50] did not detect any saxitoxins in the mantle of several bivalve species.

#### 2.5.3. Gills and Labial Palps

**Figure 5:**
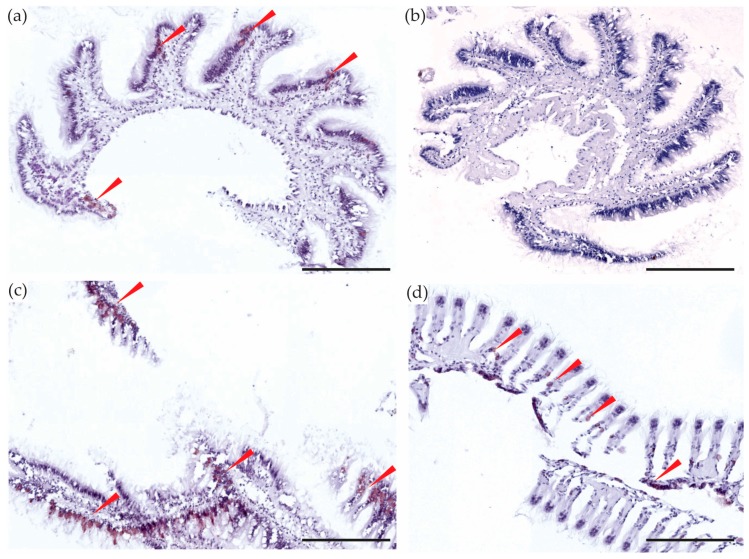
Paphies australis tissues sectioned at 7 µm. Tetrodotoxin (TTX)-specific monoclonal antibody (mAB) immunohistological staining of: (**a**) the labial palps with the TTX-mAB; (**b**) the labial palps with mAB negative control; (**c**,**d**) the gills with the TTX-mAB. TTX was identified by the brown color deposits shown by red arrows. Scale bars = 100 µm (**a**,**b**) and 50 µm (**c**,**d**).

Gills in bivalves are primarily used for respiration and feeding [49]. The pair of labial palps, situtated in the mantle cavity near the mouth, are involved in feeding. Each of the pair has two components: the inner palp is used for sorting food and transferring the selected particles to the mouth while the function of the outer palp is the production of pseudo-faeces [54]. Tetrodotoxin from an external source (e.g., food) would be expected to be present in these tissues of *P. australis*. It was visualized in the labial palps (Figure 5a) and in the gills (Figure 5c,d) of *P. australis*, explaining why TTX was detected in low concentrations in the ‘rest’ tissue group with LC-MS/MS. Other biotoxins have previously been detected in the gills of bivalves: low levels of microcystins [52] and saxitoxins [50] have been detected in gills of mussels and three species of bivalves in Palau. This result provides further evidence to support the hypothesis of a dietary source of TTX in *P. australis*.

### 2.6. Parasites

**Figure 6:**
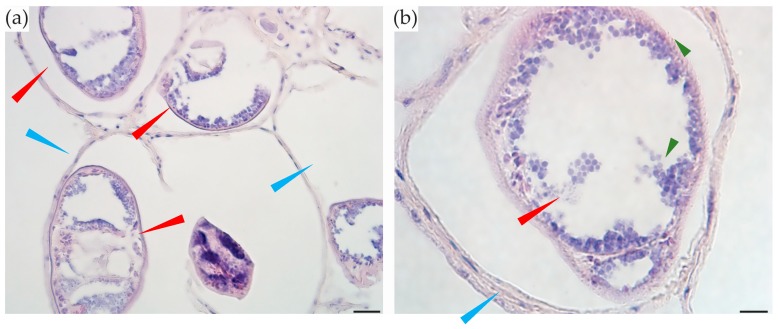
Metacercariae encysted in mantle tissues of Paphies australis. (**a**) View of a group of encysted metacercariae (red arrows) and cyst walls (blue arrows); (**b**) one metacercaria (red arrow) inside cyst wall (blue arrow); rickettsial hyperparasite colonies inside metacercaria (green arrows). Scale bars = 20 µm (**a**) and 10 µm (**b**).

Digenean trematode metacercariae (sub-adult stages) were seen (Figure 6) encysted in *Paphies australis* mantle tissues (55% of *P. australis* observed were infected). Further identification of these Gymnophallid-like worms was prevented by their poor internal condition. Most of their internal organs were missing and the body cavity of each contained many grey/purple spheres resembling colonies of rickettsia hyperparasites. No TTX was observed in the parasites in immunostained sections.

## 3. Materials and Methods 

### 3.1. Paphies australis Collection 

*Paphies australis* (*n* = 48) were collected from the Hokianga Harbour (Northland, New Zealand; 35°28′ S, 173°24′ E) on 28 September 2017 and placed in a metal shellfish collection basket. *Paphies australis* from this location had previously been shown to contain TTX (Harwood and Biessy unpub. data). Individuals were rinsed in seawater and placed in a plastic bag inside an insulated container (9–12 °C). Within two hours of collection, twelve large *P. australis* were sectioned and six small individuals (<20 mm) were kept whole and processed for immunohistochemistry as described below. Once in the laboratory (<24 h), five individuals for TTX analysis were frozen whole (−20 °C) immediately and the remaining *P. australis* (*n* = 30) were kept in an aerated aquarium (30 L), maintained at 18 ± 1 °C with a 14:10 h light:dark cycle with recirculating water for gut voidance. Bivalves were fed the microalga *Isochrysis galbana* (2 L; 12 × 10^6^ cells·mL^−1^), known to be free of TTX, every second day for nine days. The aquariums were cleaned and the water changed after seven days to maintain dissolved oxygen concentrations (7–8 mg/L) and salinity (34–35‰).

### 3.2. Sample Processing

#### 3.2.1. Sectioning for Immunohistochemistry

The twelve *P. australis* were measured (side-to-side axis) and manually shucked with a sterile blade. A longitudinal cross-sectional slice (3–5 mm) was prepared that included the stomach, adductor muscle, digestive gland, mantle, foot, gills, and gonads as described in Howard et al. [55]. Sections of siphon tissues were also prepared for histology. The six *P. australis* were left whole because of their small sizes (less than 20 mm long). Cross-sections, siphons and the small whole *P. australis* were then placed in histological cassettes (ABC Scientific, Glendale, CA, USA) and fixed in 10% formalin in seawater for 48 h. The cross-sections and siphons from the same individual were combined in one cassette. After fixation, the cassettes were transferred to 70% ethanol until further processing.

#### 3.2.2. Dissection for Tetrodotoxin Analysis

*Paphies australis* (*n* = 10) fed a TTX-free diet were collected from the aquarium after day 3, 6, and 9 and were rinsed with Milli-Q water. These, and the five that had been frozen immediately, were aseptically dissected using a sterile scalpel. The tissues were pooled into five groups (keeping each time point separate): siphon, foot, digestive gland, adductor muscles, and the ‘rest’ which included the mantle and the gonads. The pooled samples were frozen (−20 °C) for later TTX analysis using LC-MS/MS as described below.

#### 3.2.3. Immunohistochemistry

Dissected tissue samples in cassettes were then sent to Medlab (Taranaki, New Zealand) to be dehydrated, embedded in paraffin, and sectioned at 5 μm thickness. Three histological sections on individual slides were received per sample, two unstained and one ‘control’ slide stained with hematoxylin and eosin.

The following protocol was adapted from Salvitti et al. [56]. Immunohistological sections were deparaffinized in xylene and rehydrated through decreasing concentrations of ethanol (100–70%) before treatment with 3% H_2_O_2_/10% methanol to remove endogenous peroxidase activity followed by incubation with normal goat serum (VectorLabs, Burlingame, CA, USA) to prevent non-specific binding. Both the H_2_O_2_/methanol mixture and normal goat serum were diluted with 1 × Phosphate Buffered Saline (1 × PBS; pH 7.2). Slides were then incubated with a TTX-specific monoclonal antibody (mAb) T20G10 diluted to 1 μg·mL^−1^ [31] in concert with VECTASTAIN® ABC kit (VectorLabs, Burlingame, CA, USA) according to the manufacturer’s instructions (Table 1). Visualization of the antigen-antibody complex was conducted using 3,3′-diaminobenzidine (DAB)-Nickel substrate solution. Sections were counterstained with Gill’s II Hematoxylin (Sigma-Aldrich, Darmstadt, Germany) and were dehydrated using ascending grades of ethanol (60–100%), followed by two rinses in xylene for 10 min each. The slides were mounted with DPX mountant (Sigma-Aldrich), and observed using an inverted microscope (CKX41, Olympus) and photographs were taken using a slide scanner (VENTANA iScan Coreo, Tucson, AZ, USA).

Antigen–antibody complexes were visualized as brown colour deposit in positive sections and the same protocol was followed for the negative controls with the slides being incubated overnight with 1 × PBS instead of the TTX-specific monoclonal antibody.

#### 3.2.4. Tetrodotoxin Analysis

Each sample (*ca.* 0.3–3.0 g) was weighed, cut into small pieces with a sterile blade and placed in a sterile tube (50 mL) with a corresponding volume (ca. 300–3,000 µL) of Milli-Q water containing 1% acetic acid. Samples were homogenized (Ultra-Turrax^®^, IKA^®^, Wilmington, NC, USA) for 45 s to ensure complete disruption of tissues. The tubes were placed in boiling water (5 min) and then cooled in an ice bath (5 min) before briefly vortexing. Samples were centrifuged (3200× *g*, 10 min) and 0.3–1 mL of the supernatant transferred to a centrifuge tube (1.7 mL) containing 2.5–5 µL of 25% ammonia (Honeywell). Samples were then centrifuged (17,000× *g*, 1 min) and the supernatant cleaned with the GPC Solid Phase Extraction (SPE) method as described by Boundy et al. [24] using Supelclean ENVI-Carb 250 mg/3 mL SPE cartridges (Sigma-Aldrich). Tetrodotoxin was analyzed and quantified by liquid chromatography tandem-mass spectrometry analysis as described by Turner et al. [57].

#### 3.2.5. Statistical Analysis

Tetrodotoxin data from the LC-MS/MS was analyzed using R [58]. Data from the three time points (3, 6, and 9 days) were combined to enhance replicate number (after showing no significant difference) and an ANOVA with a Tukey’s post-hoc test were undertaken to determine if there are statistically significant differences in TTX concentrations among tissues and organs.

## 4. Conclusions 

Tetrodotoxin analysis of *P. australis* organs and tissues using LC-MS/MS showed that the siphons contained the highest concentrations of the biotoxin. Lower concentrations of TTX were found in the foot, digestive system, mantle, and the combined group of organs/tissues (‘rest’) which could not be individually dissected. The immunohistochemistry experiment demonstrated the micro-localization of TTX inside these organs/tissues. The toxin was primarily present in the outer layers of the siphons, rectum, digestive tubes, gills, and labial palps. Observing TTX in organs involved in feeding provides initial evidence to support the hypothesis that the source of the neurotoxin is exogenous in *P. australis*. The presence of TTX in the siphon suggests that one of its ecological roles in this species may be to reduce predation. Further studies are needed to determine if TTX migrates to other organs over time, such as those involved in reproduction, and whether the toxin is transferred to subsequent generations.

## Figures and Tables

**Table 1:** Immunohistological incubation scheme. Steps were undertaken at room temperature unless otherwise specified.

Step	Solution	Time (min)
1.	3% H_2_O_2_/10% methanol	10
2.	1 × PBS	10 × 3
3.	Normal Goat Serum	20
4.	1 × PBS	10 × 3
5.	mAB T20G10 *	Overnight at 4 °C
6.	1 × PBS	10 × 3
7.	Biotinylated secondary antibody (anti-rabbit IgG) *	60
8.	1 × PBS	10 × 3
9.	VECTASTAIN^®^ ABC reagent *	60
10.	1 × PBS	10 × 3
11.	DAB *	5–10
12.	Deionized H_2_O	5
13.	Counterstain (Gill’s II Hematoxylin)	5

PBS—phosphate buffered saline, mAB—monoclonal antibody, DAB—3,3′-diaminobenzidine. * Reagents were diluted in 1 × PBS, pH 7.2.

## Supplementary Materials

The following are available online at http://www.mdpi.com/2072-6651/10/7/282/s1, Figure S1: Observed TTX analogues from the siphon of *Paphies australis*.
