# Supplementary material for: Distribution of Tetrodotoxin in the New Zealand Clam, Paphies australis, Established Using Immunohistochemistry and Liquid Chromatography-Tandem Quadrupole Mass Spectrometry
Source: Toxins (Basel). 2018 Jul 6;10(7):282. doi: 10.3390/toxins10070282 (PMC6070791; doi:10.3390/toxins10070282)
Supplement: Supplementary file 1 [file toxins-10-00282-s001.pdf]

Supplementary Materials: Distribution of Tetrodotoxin in the New Zealand Clam, *Paphies australis,* Established Using Immunohistochemistry and Liquid Chromatography-tandem Quadrupole Mass Spectrometry

Laura Biessy, Kirsty F. Smith, Michael J. Boundy, Stephen C. Webb, Ian Hawes and Susanna A. Wood

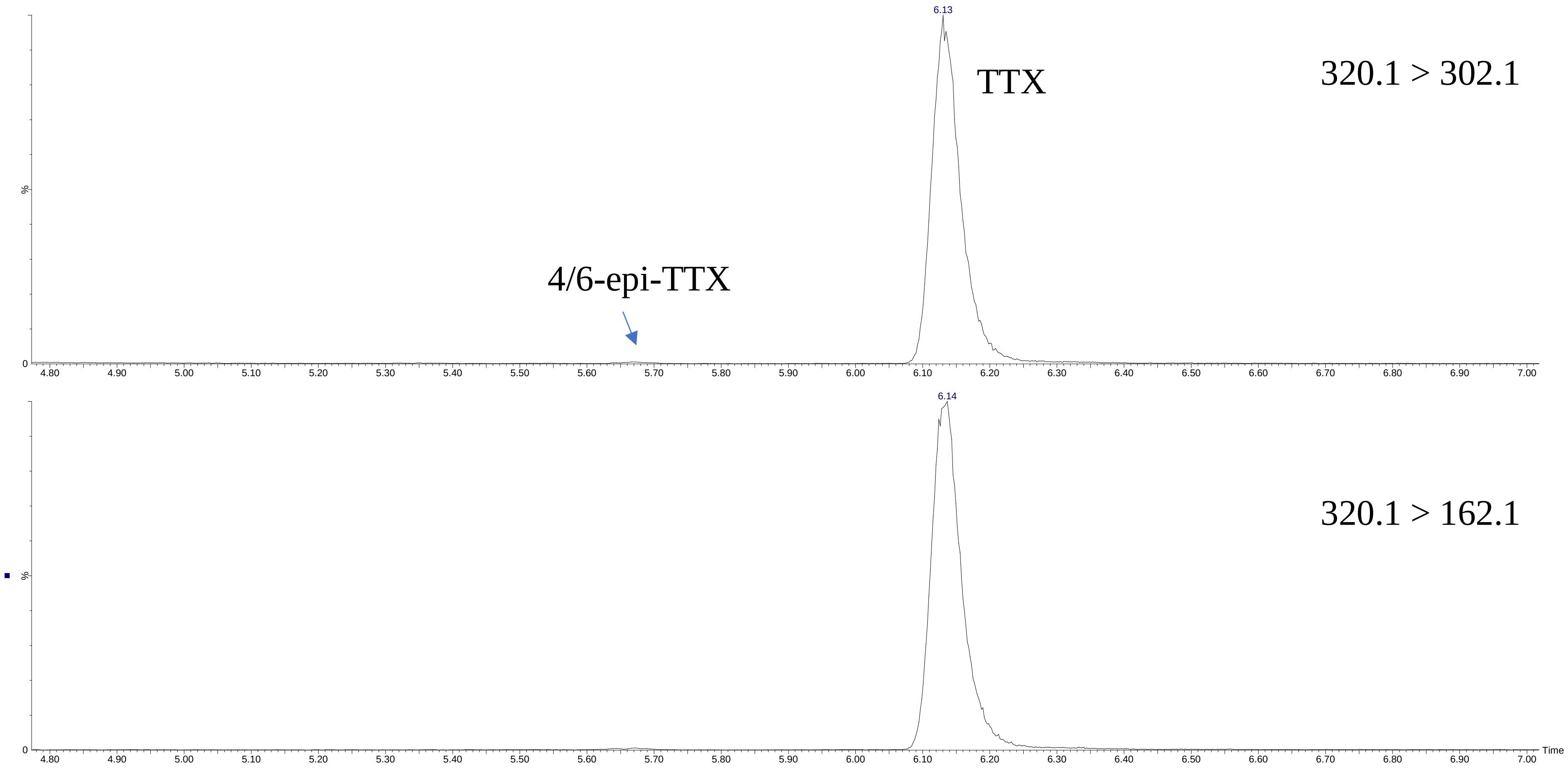

6.13
TTX
320.1 > 302.1
4/6-epi-TTX
%
0
4.80
4.90
5.00
5.10
5.20
5.30
5.40
5.50
5.60
5.70
5.80
5.90
6.00
6.10
6.20
6.30
6.40
6.50
6.60
6.70
6.80
6.90
7.00
6.14
320.1 > 162.1
Time

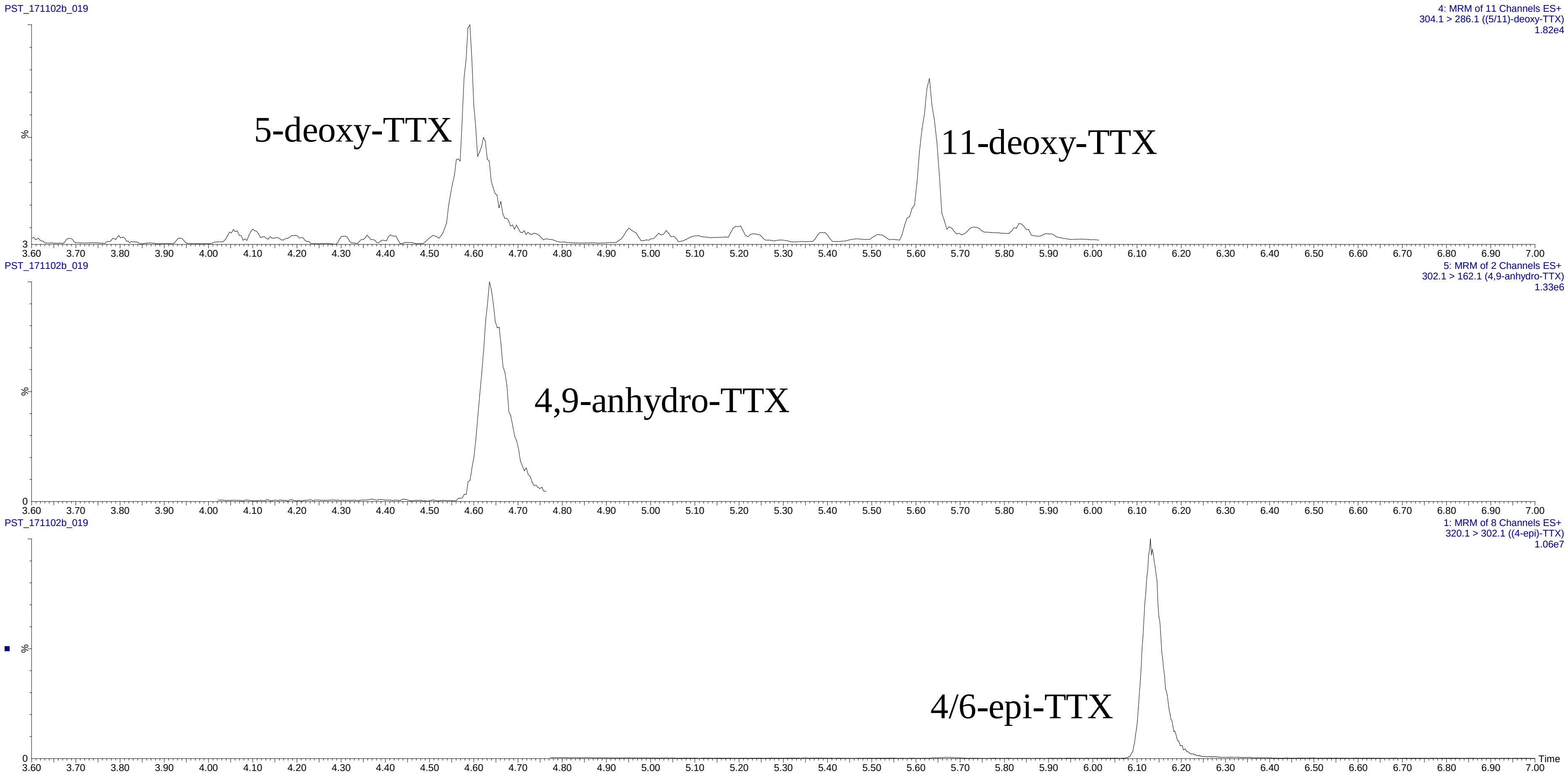

Figure S1. Observed TTX analogues from the siphon of *Paphies australis.*
